# Are You PREPAREd? A Structured Approach to Support Safe and Confident Transitions to After-Hours Care

**DOI:** 10.5334/pme.2504

**Published:** 2026-10-01

**Authors:** Anique Baten, Inge A. Pool, Jesse Lammerts, Chantal P. Rovers, Cornelia R. M. G. Fluit, Jacqueline de Graaf

**Affiliations:** 1 Department of Research in Learning and Education, Radboud university medical center Health Academy, Nijmegen, The Netherlands; 2 Isala Academy, Isala Hospital, Zwolle, The Netherlands; 3 Gelre Academy, Gelre Hospitals, Apeldoorn, The Netherlands; 4 Department of Intensive Care, Isala Hospital, Zwolle, The Netherlands; 5 Department of Internal Medicine, Radboud university medical center, Nijmegen, The Netherlands; 6 Division of Postgraduate Medical Education, Radboud university medical center Health Academy, Nijmegen, The Netherlands

## Abstract

**Background and Need for Innovation:**

Early after-hours work represents a demanding transition for newly graduated doctors. Preparation is often fragmented, with limited attention to the integrated clinical, organisational, and supervisory demands of after-hours care, contributing to uncertainty that may affect both patient safety and professional development.

**Goal of Innovation:**

PREPARE (PREParing for After-hours caRE) is an eight-week programme designed to support new graduates during their early postgraduate transition by enabling safe and confident participation in after-hours care.

**Steps Taken for Development and Implementation:**

PREPARE was developed through a research-informed, iterative design process. The programme integrates five interconnected components—familiarisation, workplace learning, structured teaching, guided reflection, and entrustment—within a coherent framework that runs parallel to routine clinical work. Rather than functioning as a fixed curriculum, PREPARE provides a flexible structure that aligns educational activities, workplace learning, reflection, and entrustment, while allowing adaptation to local service contexts.

**Evaluation of Innovation:**

Formative evaluation throughout development and early implementation focused on perceived value, feasibility, and alignment with after-hours responsibilities. New graduates and supervisors reported increased confidence, improved preparedness for managing uncertainty, and enhanced understanding of interprofessional collaboration.

**Critical Reflection on Our Process and Innovation:**

Reflection highlighted how PREPARE engages with real clinical complexity and exposes tensions between educational ambition and organisational feasibility. While the programme demonstrates how after-hours transitions can be more deliberately supported, its sustainability and transferability depend on organisational commitment and protected time for learning.

## Background and Need for Innovation

Transitioning from daytime clinical work to increasingly independent on-call work—particularly night shifts—represents one of the most complex and consequential milestones for newly graduated doctors. Research shows that new graduates often feel insufficiently prepared for this transition [1,2,3]. The consequences of these feelings extend well beyond individual discomfort: perceived inadequate preparedness can lead to stress and impaired performance, thereby compromising patient safety [4,5,6]. Conversely, when adequately supported, after-hours shifts—through exposure to unfamiliar, higher-urgency clinical scenarios alongside increased responsibility—may accelerate professional and personal development [7,8].

In the Netherlands, new graduates enter clinical practice immediately after graduation and are formally licensed to perform basic clinical tasks from day one. This transition often involves starting work in a new hospital or department, typically supported by a variably structured orientation period focused on local systems, workflows, logistics, and technical skills such as basic life support. New graduates usually begin participating in evening or night shifts after two to three months of clinical work, entering a setting characterised by higher urgency, reduced staffing, and predominantly indirect supervision. While the organisation and timing of this transition vary across countries, the short run-up to first on-call shifts and accompanying increase in responsibility are widely recognised challenges [1,2].

Findings from the programme of research underpinning PREPARE—including longitudinal and qualitative studies of new graduates’ early on-call experiences—revealed several systemic gaps that make the first shift unnecessarily difficult. Onboarding typically centres on daytime ward routines, with supernumerary time often limited by staffing pressures. Learning activities tend to be fragmented, targeting isolated skills rather than the integrated competence required to function effectively on call. Opportunities for structured reflection vary considerably, as do the form and availability of mentorship. Moments of direct observation are restricted by the tension between service demands and educational intentions. Finally, entrustment decisions remain largely implicit, representing a missed opportunity for building self-efficacy. These findings mirror international literature, demonstrating that insufficient preparation for after-hours care and related tasks is a recurring challenge across healthcare systems [1,2,9].

To better support newly graduated doctors during the transition into after-hours work, we developed PREPARE (PREParing for After-hours caRE): an eight-week programme positioned in the early postgraduate phase, prior to participation in on-call work, bringing together five interconnected components—familiarisation, workplace learning, structured teaching, guided reflection, and entrustment—within a coherent, practice-embedded structure to support early participation in on-call care.

## Goals of Innovation

The primary aim of PREPARE was to enable newly qualified doctors to begin their first after-hours shifts safely and with confidence, by supporting a gradual progression towards the responsibilities inherent in after-hours care during the early postgraduate phase. A second aim was to support the educational potential of on-call shifts, which is currently underused despite their substantial experiential learning value.

## Steps Taken for Development and Implementation of Innovation

PREPARE resulted from a multi-year research and design trajectory that spans foundational studies, evolving pedagogical insights, international literature, iterative testing and practice-based refinement, and recent implementation (Figure 1). These phases are outlined below, together with the key insights that shaped the programme’s eventual form.

**Figure 1:**
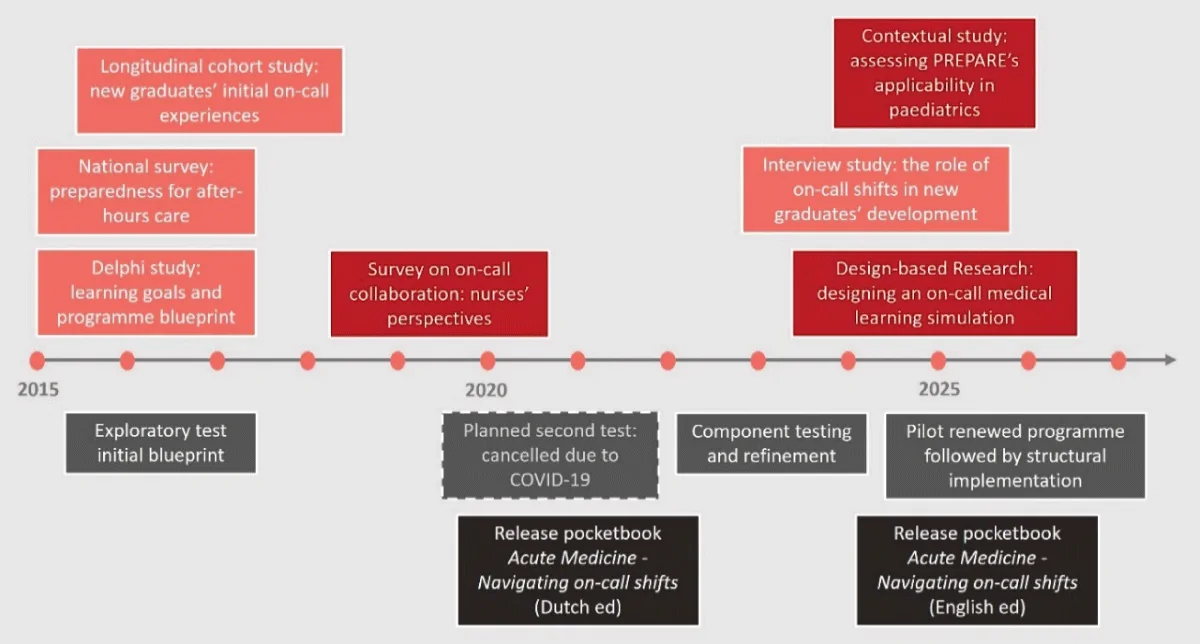
Multi-year development trajectory underpinning the PREPARE programme. *Note:* Above the timeline: PhD studies (pink) and supervised student and resident projects (red). Below the timeline: practice-based development activities (light grey) and programme outputs (dark grey).

### Pedagogical and Theoretical Foundations

PREPARE was designed to cultivate adaptive expertise in newly graduated doctors during early on-call work, prioritising their ability to function effectively in unfamiliar, dynamic, and high-pressure situations [10]. Rather than attempting exhaustive preparation for all possible scenarios, the programme supports learners in progressively engaging with increasing clinical complexity and developing tools that enable action across a range of unpredictable situations. Earlier empirical work identified generic learning objectives (Appendix A) and recurring challenges in early after-hours care. These insights informed a shared framework that progresses from technical skills to non-technical skills (NTS) and, finally, basic didactic skills, while providing sufficient flexibility to accommodate local clinical contexts.

Technical skills are addressed first, reflecting the need for concrete guidance early in the transition [11]. NTS are subsequently introduced through authentic after-hours challenges such as communication under pressure, situational awareness, prioritisation, and help-seeking behaviour. Drawing on Flin’s work on NTS [12] and Edmondson’s concept of psychological safety [13], this first phase emphasises effective teamwork, shared situational awareness, and a learning climate in which uncertainty can be openly discussed. Tools such as the ABCDE framework (Airway, Breathing, Circulation, Disability and Exposure) and anticipatory planning support clinical reasoning and flexible action in the face of uncertainty. Finally, participants consolidate learning through supervising medical students and gradually developing basic didactic skills.

Simulation was used as a translational strategy to support transfer between educational activities and clinical practice [14]. The SimZones framework informed the alignment and sequencing of learning activities across different simulation modalities [15].

### Development process

The research and design trajectory began with a national survey, which demonstrated that on-call preparedness is strengthened by meaningful early clinical exposure, structured teaching, and assessment practices aligned with after-hours responsibilities [3]. A subsequent three-round Delphi study refined these insights by articulating generic learning objectives for safe after-hours care and identifying the educational and organisational support required to achieve them (Appendix A). Together, these findings informed the programme’s first blueprint, which was explored through pilot activities with newly graduated doctors and interprofessional learners. Participant feedback highlighted the need for a more explicitly programmatic structure, stronger interprofessional integration, and learning resources that were more readily applicable in clinical practice. These insights informed the development of a concise pocketbook, later released in both Dutch and English editions to support everyday use during after-hours work [16].

In parallel, a longitudinal cohort study conducted as part of the PREPARE development trajectory explored how newly graduated doctors were prepared for on-call work and which challenges they encountered despite existing preparation efforts. Insights from this work highlighted substantial variation in preparation pathways across hospitals, persistent gaps related to non-technical skills, and the importance of deliberate exposure to the urgency and workload pressures inherent to after-hours care. Participants also highlighted the challenges of supervising medical students during demanding shifts. Additional work on interprofessional collaboration informed the refinement of programme resources and the development of an on-call simulation tool addressing prioritisation, common clinical queries, and maintaining oversight. These findings informed subsequent refinements to programme content and learning activities and highlighted the importance of considering organisational factors such as staffing, supervision, and protected time.

Although further testing was interrupted by the COVID-19 pandemic, programme refinement continued across multiple learner groups, ranging from medical students to experienced multidisciplinary teams. These activities informed adjustments to programme content, sequencing, feasibility, and the iterative refinement of the on-call simulation tool, which is now structurally embedded within PREPARE. Additional insight came from a qualitative interview study conducted as part of the PREPARE development trajectory, which explored how first after-hours shifts contribute to new graduates’ learning and how supervisors shape that learning [17]. This work suggested that first shifts can stimulate development across multiple educational domains and that adequately supported early exposure to after-hours responsibilities may be experienced as valuable rather than premature.

The fully renewed eight-week programme was piloted in late 2024 in a large teaching hospital with seven newly qualified doctors and one final-year medical student. Interprofessional teaching sessions were facilitated through alignment with an existing start-phase programme for newly graduated nurses. Participating new graduates reported high satisfaction and increased perceived on-call preparedness, confirming the value of the programme revisions made across design and learning resources. These evaluations informed a final round of minor refinement, after which the programme received institutional endorsement and was structurally adopted.

### Programme overview

PREPARE is delivered over eight weeks through five interconnected components that support new graduates during their early postgraduate transition by integrating workplace learning, structured education, guided reflection, and assessment alongside routine clinical work. The programme runs parallel to clinical duties rather than replacing service delivery, supporting safe participation in after-hours care while strengthening professional and personal development. Figure 2 provides an overview of the programme structure.

**Figure 2:**
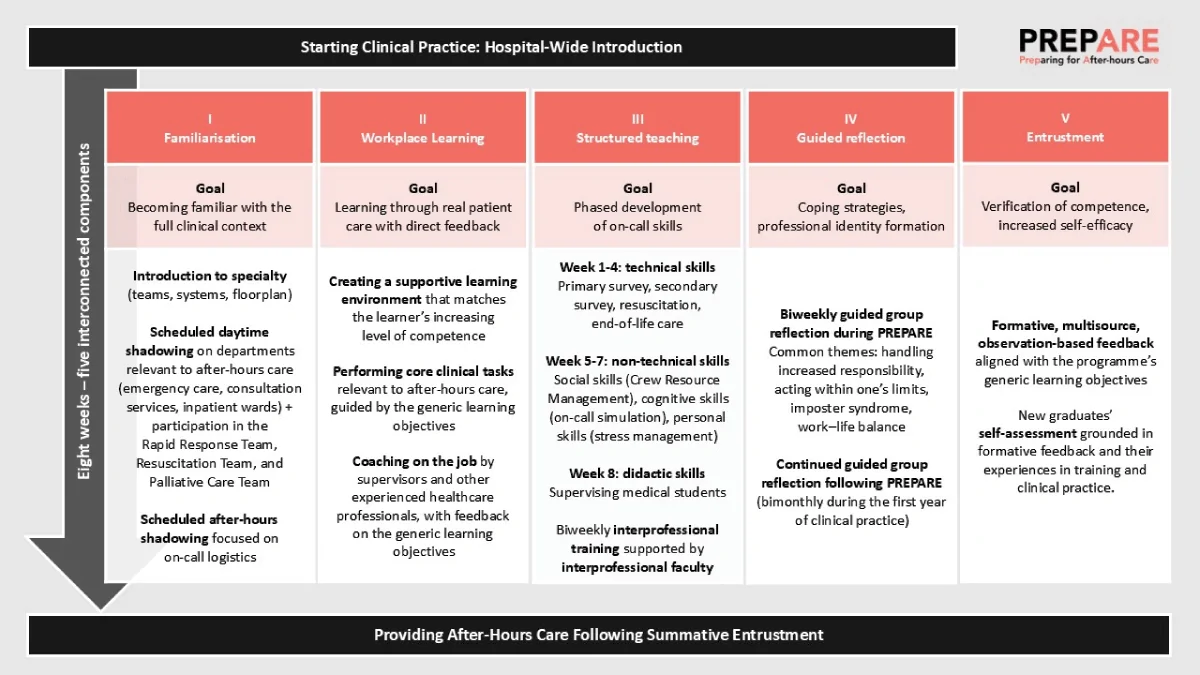
Overview of the five interconnected components of PREPARE.

Through daytime and after-hours shadowing, participants become familiar with the people, systems, logistics, and environments central to on-call care. Guided by the generic learning objectives outlined in Appendix A, they then engage in core clinical tasks relevant to after-hours care, supported by workplace coaching and feedback from supervisors and other experienced healthcare professionals.

Structured teaching complements workplace learning by addressing both technical and non-technical skills, with selected sessions delivered in interprofessional formats that reflect the collaborative nature of after-hours care. Guided reflection supports participants in consolidating learning, contextualising challenges, and exploring emerging aspects of professional identity. Entrustment serves as the integrative endpoint of PREPARE, with readiness for independent after-hours work determined through workplace observations, multisource feedback, and participants’ self-assessment.

PREPARE gains additional strength through deliberate alignment with existing educational structures. These connections enrich learning opportunities while supporting implementation and sustainability by building on expertise and infrastructure already embedded within the organisation. For example, interprofessional sessions were linked to the hospital’s start-phase programme for newly graduated nurses, while collaboration with the undergraduate curriculum enabled participation in simulation activities and near-peer teaching. Experienced residents further contributed as instructors, enhancing training quality while developing their own educational skills.

## Evaluation of Innovation

Evaluation occurred throughout development and implementation, using facilitated group evaluations, routine post-session feedback, and periodic instructor reviews to assess perceived value, feasibility, alignment with after-hours responsibilities, and fidelity to the programme’s core design. During exploratory testing, participants consistently emphasised the programme’s practical relevance and the clarity it provided about early after-hours work.

The 2024 pilot of the fully renewed eight-week programme further underscored its contribution to early readiness. New graduates reported increased confidence, greater preparedness for managing uncertainty, and improved understanding of interprofessional collaboration. New graduates also highlighted the value of the programme’s attention to non-technical skills, stress regulation, prioritisation, and maintaining oversight during acute clinical work. Supervisor observations closely mirrored these reports. As one neurology supervisor noted:

*“Even though the programme has only been running for a short time, our newly graduated doctors seem to start their on-call shifts with more confidence and work more systematically during shifts. Of course, it’s a small sample, but the feedback I receive is unanimously very positive across all aspects.”*

Following implementation, a quality improvement project explored the applicability of PREPARE within paediatrics. The programme was perceived as relevant in this setting and required only minor adaptations, primarily the inclusion of a broader range of paediatric cases.

Across all phases, evaluations were intentionally formative, focusing on perceived value, feasibility and early indicators of improved preparedness. Outcomes such as workplace performance and system-level effects were considered beyond the scope of this developmental stage [18]. Now that the programme has moved beyond its initial development phase, more outcome-oriented evaluation represents an important next step. Growing interest from other hospitals suggests opportunities for future multicentre evaluation to examine these domains more systematically.

## Critical Reflections on our Process and Innovation

In this section, we reflect on how pedagogical choices, contextual realities, and organisational conditions shaped PREPARE and what these experiences reveal about supporting transitions into after-hours practice.

### Reflecting on the Development Process

Developing PREPARE across multiple years created space for repeated cycles of observation, testing and refinement; it also meant that early insights required periodic re-examination. In retrospect, adopting a more formalised design-based research structure from the outset might have enabled shorter iteration cycles and earlier articulation of core design principles. Nevertheless, later studies consistently reproduced the patterns identified early on—variation in preparation pathways, persistent gaps in training initiatives, insufficiently effective clinical orientation, and underused learning potential—suggesting that these challenges reflect structural features of early clinical practice rather than time-bound shortcomings.

A second, recurring tension concerned the balance between pedagogical ambition and organisational feasibility. PREPARE’s emphasis on authenticity required access to simulation facilities, cross-departmental coordination, and substantial instructor availability—resources that are not always readily accessible in a service-driven environment. While the overall investment per cohort is manageable (approximately 140 instructor hours for a group of ~20 participants over an eight-week period), implementation repeatedly raised organisational questions regarding responsibility and resourcing: who releases staff from clinical duties, and through which budget is protected time allocated? As the director of education of the implementing hospital noted:

*“The content is supported, but the discussion is about the money — and that is a broader risk within hospitals.”*

This experience underscored that the sustainability of programmes such as PREPARE depends not only on educational value, but on organisational structures that enable time, coordination, and shared ownership, as well as a willingness to invest in these conditions [19]. While small-scale evaluation and qualitative feedback provided insight into mechanisms of impact, modest samples limit claims about broader outcomes and highlight the need for more systematic, longitudinal evaluation. At this developmental stage, however, the programme’s value may lie less in demonstrable downstream outcomes than in its capacity to address well-documented vulnerabilities of early after-hours practice.

### Reflecting on the PREPARE Programme

Building on our experiences with PREPARE, several insights emerged that may be relevant for supporting transitions into after-hours practice. Our experience reinforced the importance of engaging learners with the complexity of clinical practice rather than relying on simplified approximations. Attempts to ‘protect’ learners by reducing complexity risk undermining transfer and constraining the development of adaptive expertise [10]. PREPARE embraced this complexity but introduced it in a deliberate, phased manner—from technical skills to non-technical skills and ultimately more complex clinical application—allowing new graduates to gradually assume authentic responsibility and become increasingly equipped to manage complexity and uncertainty in clinical practice. This also highlighted the importance of understanding the realities of after-hours work within the local clinical context, including task demands, responsibility distribution, and available support structures.

A second insight concerned the value of maintaining structural clarity while allowing contextual flexibility. Variation in workflows, staffing models, and service demands renders rigid, standardised approaches impractical. PREPARE therefore functions as a flexible framework rather than a fixed curriculum, enabling alignment with existing structures. Importantly, this flexibility does not imply informality: it requires explicit choices about which design principles are essential and which implementation features can vary by context.

A third insight concerned the positioning of structured support for after-hours work within the educational continuum. Although the timing of first night shifts varies considerably across settings, previous work suggests that successful transitions depend not only on timing but also on the availability of graduated responsibility and appropriate support structures [20]. Our findings and implementation experiences suggested that, regardless of timing, preparedness depends less on when after-hours work begins and more on whether new graduates are adequately prepared for the tasks and responsibilities involved and are familiar with the clinical environment, including escalation pathways and available support.

In some contexts, such preparation may be embedded within undergraduate education. In the Dutch context, limited opportunities for sustained after-hours participation during undergraduate training and frequent transitions to a different hospital or specialty make early postgraduate support both pragmatic and educationally meaningful. However, positioning structured support within clinical practice also brings specific organisational challenges. Once education is embedded in routine service delivery, programmes become inherently dependent on local staffing, scheduling, supervisory capacity, and institutional priorities. Our experience showed that organisational commitment is therefore not merely enabling, but essential for sustainable implementation.

### Future directions

Future development will focus on deeper workplace embedding so that the principles underlying PREPARE are consistently reinforced by supervisors and clinical teams. Our experience suggests that supporting early after-hours work requires a shared organisational commitment rather than relying solely on individual learners. As implementation expands across departments and settings, opportunities emerge for more systematic evaluation of PREPARE’s educational and organisational impact.

## Additional File

The additional file for this article can be found as follows:

10.5334/pme.2504.s1 Appendix A. Delphi Study Informing the PREPARE Programme Blueprint.
