# Supplementary material for: Are You PREPAREd? A Structured Approach to Support Safe and Confident Transitions to After-Hours Care
Source: Perspect Med Educ. 2026 Oct 1;15(1):1050–7. doi: 10.5334/pme.2504 (PMC13629528; doi:10.5334/pme.2504)
Supplement: Supplementary file 1 [file pme-15-1-2504-s1.pdf]

Appendix A. Delphi Study Informing the PREPARE Programme Blueprint

To inform the development of PREPARE, we conducted a three-round Delphi study involving newly graduated doctors and experienced healthcare professionals. The study explored which competencies and support structures were considered important for preparing new graduates for after-hours care. Using principles from deliberate practice as a sensitising framework, participants identified both the capabilities required for safe participation in after-hours work and the conditions needed to support their development.

Consensus was reached on a set of generic learning objectives spanning clinical management, communication, teamwork, prioritisation, decision making, and recognising one's own limitations. Participants emphasised that preparedness for after-hours care extends beyond technical competence and requires the ability to function effectively under pressure, collaborate across professional boundaries, and seek support appropriately. These findings informed the generic learning objectives that underpin workplace learning, structured teaching, feedback, and assessment within PREPARE.

The study also identified educational and organisational conditions that support learner development, including meaningful workplace participation, gradual exposure to responsibility, direct observation and feedback, access to experienced supervisors, and explicit attention to progression towards independent practice. These findings informed several core design principles of PREPARE, including familiarisation, workplace learning, guided reflection, and entrustment. The consensus findings are summarised in Tables A1 and A2. While these findings provided an important foundation for the programme blueprint, the final design of PREPARE was further shaped through subsequent research, piloting, stakeholder consultation, and implementation experiences.

Table A1. Generic on-call Learning Objectives

The new graduate…

- Demonstrates practical knowledge and skills relevant to common after-hours presentations, including the use of local protocols.*
- Recognises and manages the acutely ill patient, providing appropriate initial stabilisation until care is handed over.
- Identifies circulatory arrest and initiates resuscitation according to current guidelines.
- Requests and interprets commonly used diagnostic tests (X-ray, ECG, laboratory investigations).
- Communicates clinical information concisely and structured, ending with a clear question or management proposal.
- Makes timely, responsible decisions within their scope of practice and escalates appropriately when needed.
- Maintains situational awareness and prioritises tasks according to urgency.

Table A1. Generic on-call Learning Objectives (continued)
**The new graduate…**<br>• Communicates effectively with patients (information-giving, shared decision-making) and colleagues (instruction, escalation, seeking help).<br>• Works effectively within multidisciplinary teams and, when required, coordinates team actions while acknowledging others' roles and expertise.<br>• Provides structured handovers to safeguard continuity of care.<br>• Applies these competencies under time pressure and in unpredictable circumstances.
*The context-specific translation involves tailoring these generic after-hours care learning objectives to the realities of local practice. This includes specifying which presentations, conditions, clinical skills, workflows, and supervisory arrangements are relevant within a given specialty and hospital setting. Such contextualisation is required for every specialty in which newly qualified doctors provide after-hours care.

Table A2. Orientation components and formal training topics supported by expert consensus[a]

Orientation	*Mean ± SD*	*% ≥6*
Clinical placements in relevant departments (e.g. Accident & Emergency (A&E), consultation services, associated hospital wards)	6.4 ± 0.6	94.9
Supernumerary placement during orientation	6.3 ± 0.8	82.1
Familiarising with hospital logistics	6.2 ± 1.1	87.2
Integrating structured direct supervision into orientation	6.1 ± 1.1	79.5
Structured teaching sessions	*Mean ± SD*	*% ≥6*
Basic Life Support	6.5 ± 1.1	94.8
ABCDE-methodology	6.4 ± 0.9	84.6
Common (acute) pathology (incl. relevant protocols)	6.4 ± 0.7	92.3
EKG interpretation	6.1 ± 1.2	77.0
Appendix: [a] Scores ranked high – low.		
